# Transitioning from a COMS‐based plaque brachytherapy program to using eye physics plaques and plaque simulator treatment planning system: A single institutional experience

**DOI:** 10.1002/acm2.13902

**Published:** 2023-01-13

**Authors:** Sheridan G. Meltsner, Anna Rodrigues, Miguel A. Materin, David G. Kirsch, Oana Craciunescu

**Affiliations:** ^1^ Department of Radiation Oncology Duke University Durham North Carolina USA; ^2^ Departments of Ophthalmology Duke University Durham North Carolina USA

**Keywords:** commissioning, Eye Physics eye plaques, ocular brachytherapy, plaque brachytherapy, Plaque Simulator treatment planning system

## Abstract

The aim of this work is to describe the implementation and commissioning of a plaque brachytherapy program using Eye Physics eye plaques and Plaque Simulator treatment planning system based on the experience of one institution with an established COMS‐based plaque program. Although commissioning recommendations are available in official task groups publications such as TG‐129 and TG‐221, we found that there was a lack of published experiences with the specific details of such a transition and the practical application of the commissioning guidelines. The specific issues addressed in this paper include discussing the lack of FDA approval of the Eye Physics plaques and Plaque Simulator treatment planning system, the commissioning of the plaques and treatment planning system including considerations of the heterogeneity corrected calculations, and the implementation of a second check using an FDA‐approved treatment planning system. We have also discussed the use of rental plaques, the analysis of plans using dose histograms, and the development of a quality management program. By sharing our experiences with the commissioning of this program this document will assist other institutions with the same task and act as a supplement to the recommendations in the recently published TG‐221.

## 1 INTRODUCTION

Ocular melanoma is a malignant tumor of the melanocytes that occurs mostly in the choroid, ciliary body, and iris and is commonly treated using radioactive plaque brachytherapy.^1^ The large‐scale Collaborative Ocular Melanoma Study (COMS) standardized the use of I‐125 COMS plaques, which are comprised of gold alloy shells and Silastic inserts for seed localization.^2^ The specifics of the COMS planning protocols and the outcome of the study are well established.^3^ Other plaques have been developed for use in plaque brachytherapy,^4^, ^5^, ^6^, ^7^, ^8^, ^9^ including the Eye Physics (EP) plaques developed by Eye Physics, LLC (Los Alamitos, CA)^10^, ^11^ and available from IsoAid, LLC (Port Richey, FL) which have been in clinical use in a preliminary form since the early 1980s^12^ and in their current form since the early 1990s.^13^, ^14^ EP plaques are gold alloy with collimated slots into which radioactive seeds are glued, negating the need for inserts. The plaques are available in a variety of shapes and the collimation affects the intraocular radiation distribution. The Plaque Simulator (PS) treatment planning system (TPS) is a 3D treatment simulation and modeling package for plaque brachytherapy of ocular tumors (Eye Physics, LLC, Lost Alamitos, CA). The PS TPS utilizes a Task Group 43 (TG‐43) based dose calculation algorithm and also includes dose calculation options including correcting for heterogeneities and backscatter from the gold alloy plaque.^11^ All calculations are performed in water. Neither the EP plaques nor the PS TPS are FDA approved.

Prior to November 2016, our institution used a COMS‐based plaque brachytherapy program, treating approximately 450 patients between 1997 and mid‐2016. Primary dose calculations were performed in BrachyVision (BV) (Varian Medical Systems, Palo Alto, CA) using a TG‐43‐based line source model and an in‐house point source‐based spreadsheet was used as an independent secondary dose calculation. Both primary and secondary calculations assumed a homogeneous water phantom and did not account for the Silastic insert or the gold alloy plaque. Rather than prescribing the COMS‐standard of 80 Gy, the default prescription was 70 Gy based on prior retrospective studies.^15^, ^16^, ^17^

At the request of a new ocular melanoma surgeon, our group was tasked to implement the transition from COMS to EP plaques. The surgeon requested this because the EP plaques are thinner than the COMS plaques, which allows procedures to be more efficient because placement is easier and requires less cutting of eye muscles as the plaque will often fit underneath the muscle. The thinner plaque also allows for increased patient comfort throughout the duration of the insertion. Due to the more realistic dose calculations in PS because of the implementation of heterogeneity corrections, it was also decided to commission the PS TPS to use with the EP plaques. At the time, there was a lack of published guidelines on how to implement such a transition. TG‐129 was available but focused more on dosimetric considerations of eye plaques.^18^ Since the commissioning of our program, the AAPM has published TG‐221 which more specifically recommends practices for plaque programs and gives general guidelines for such programs.^19^ Much of our commissioning process performed prior to its publication followed their subsequent recommendations. Thus the purpose of this paper is to present a single institution implementation of a new plaque brachytherapy program.

Our process for implementing a new program using Eye Physics plaques and Plaque Simulator treatment planning system involves the following steps which will be discussed in detail.

1 Address FDA approval of treatment planning system and plaques
2 Comments on commissioning IsoAid IAI‐125A seed model in BrachyVision and Plaque Simulator
3 Commission Eye Physics plaques
3.1 Plaque construction and consistency considerations
3.2 Plaque commissioning in BrachyVision
4 Commission Plaque Simulator treatment planning system
4.1 Plaque Simulator acceptance testing
4.2 Configuring plaque simulator software
4.3 Plaque Simulator commissioning of considered Eye Physics plaques
4.4 Plaque Simulator eye model, image‐based planning, RDAH/DVH, and prescription considerations
5 Quality assurance of plaque therapy program
5.1 Establishing an independent second dose check
5.2 Independent seed assay program and pre‐loaded and pre‐sterilized plaque considerations
5.3 Creating procedures for program maintenance, upgrades, improvements, and data accessibility
5.4 Quality program management
6 Procedures and workflow

### 1.1 Address FDA approval of treatment planning system and plaques

Guidelines on the use of non‐FDA approved eye plaques are provided by AAPM Task Group 167 Guidelines by the AAPM and GEC‐ESTRO on the use of innovative brachytherapy devices and applications. TG‐167 recommends that “institutions using these [non‐FDA approved] devices must either obtain IRB approval or request the manufacturer to obtain FDA approval.”^20^ The subsequent AAPM Task Group 221 “AAPM recommendations on medical physics practices for ocular plaque brachytherapy,” which was published after our transition to EP plaques and the PS TPS, also discusses this issue. While encouraging the use of heterogeneity corrections in eye plaque calculations, TG‐221 does acknowledge the lack of available FDA‐approved devices and treatment planning systems that perform such calculations. It encourages vendors to seek FDA approval, but also allows for the use of such software stating that the clinical physicist “must appropriately commission and validate dose calculations.”^19^

FDA approval of medical devices was not established in the United States until 1976. The COMS plaques were in use before 1976, so they did not require FDA approval, nor did the original dose calculation implementation.^20^ However, the lack of FDA approval of EP plaques and the PS TPS may be an issue when transitioning away from COMS plaque and therefore should be explicitly addressed.

As noted in TG‐221, neither the EP plaques nor the associated PS TPS software are FDA approved. The manufacturer invokes FDA approval exemption due to their designation as Class I devices, as such devices (i.e. “A manual radionuclide applicator system…includ[ing]…treatment planning computer programs”) are exempted from FDA approval as per Title 21: Sec. 829.5650.^21^ However, at the time of commissioning, the company was not seeking FDA approval.

At our institution, the FDA approval issue was addressed by forming a multidisciplinary panel of senior faculty within the Radiation Oncology department to review the EP plaques and PS TPS, to compare them to COMS plaques and prior treatment planning approach, and consider whether to approve the EP plaques and PS TPS for use. This group included the chair of the radiation oncology department, the director of the physics division, the radiation oncologist primarily responsible for the plaque brachytherapy program, the ocular surgeon who performs the procedures, and the members of the brachytherapy physics team. A letter summarizing the results and findings of our commissioning process, including the intention to continue using BrachyVision, an FDA‐approved TPS, as an independent second dose check, was presented to this group. The intention was to include both PS and BV reports in the patient's electronic medical record. Although FDA approval for EP plaques or PS TPS was not present or forthcoming, after reviewing the available information the faculty panel agreed upon the use of PS TPS and the EP plaques as defined by our commissioning documents.

Our decision to clinically use this plaque and planning system was also influenced by other institutions, as the PS TPS and the EP plaques have been used extensively in the field since 1990,^13^, ^14^ despite the lack of FDA approval. There have been a number of papers published on the use of these plaques in clinical practice without any explicit discussion about FDA approval.^13^, ^14^, ^22^, ^23^ As far as the authors are aware, this is the only publication to specifically address how we approached the use of these plaques at our institution.

### 1.2 Comments on commissioning IsoAid IAI‐125A seed model in BrachyVision and plaque simulator

The IsoAid IAI‐125A seed model was commissioned in both BV and PS using institutional protocols following AAPM recommendations made in TG‐56, TG‐43, and TG‐53.^24^, ^25^, ^26^ This step may be performed by other institutions following their own procedures but as it is not unique to the eye plaque brachytherapy program and is, rather, simply a process related to any new LDR seed model, we will not discuss it in detail in this publication.

### 1.3 Commissioning eye physics plaques

The amount of gold alloy and the more precise and involved manufacturing process required due to the design of the EP plaques means that they are significantly more expensive than COMS plaques. Indeed, according to the vendor, they know of no institution that maintains their own inventory of EP plaques. Instead, institutions rent EP plaques on a per‐patient basis. Switching our program to renting preloaded plaques does mean that we cannot physically commission every plaque being used clinically, so we needed to be confident that the plaque manufacture procedures lead to consistent plaques. To this end, we investigated the construction of the plaques as well as reviewed publications that investigated the actual physical plaques. Last, in order to use the FDA approved BrachyVision (BV) treatment planning system for independent secondary dose check calculations, we commissioned the EP plaques in BV.

#### 1.3.1 Plaque construction and consistency considerations

**FIGURE 1:**
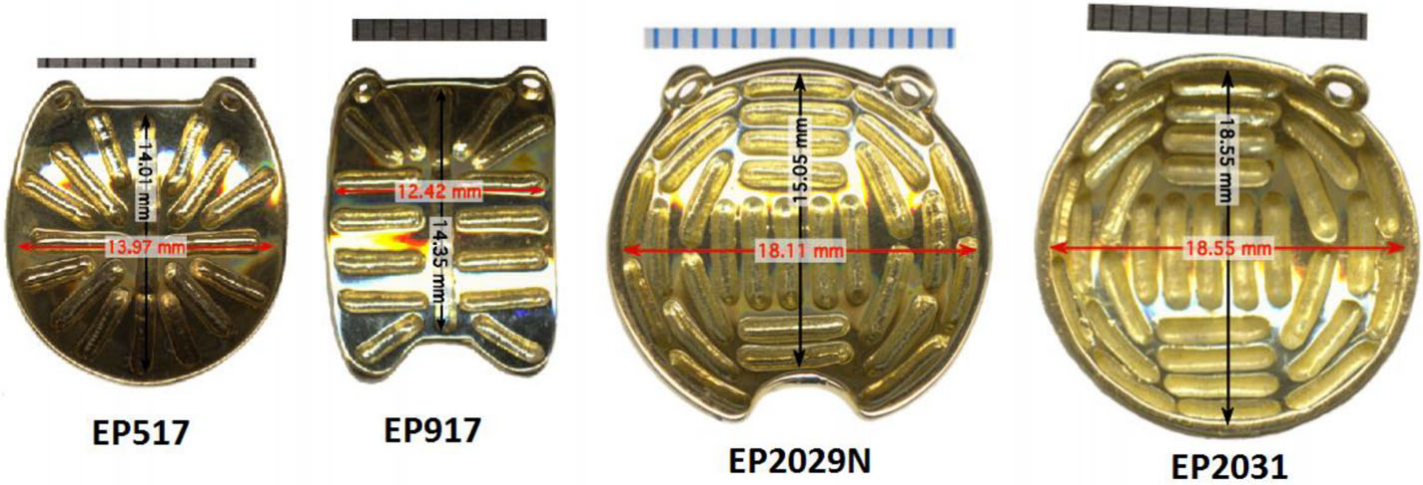
Images of the four Eye Physics plaques initally commissioned (https://eyephysics.com/)

As discussed previously, the Eye Physics (EP) plaques use collimated slots inherent in the design of the plaque, rather than Silastic carriers, to reproducibly secure the seeds and collimate the radiation. Several plaque examples can be seen in Figure 1. Second generation plaques are cast in a gold alloy using the original molds made from wax prototypes in the 1980s and 1990s. Third generation plaques are cast directly from 3D prints, and the seed locations specified in PS come directly from these 3D print files.^27^ Aryal et al. published their comparison of the seed coordinates in PS to those manually measured from a physical EP917 (2nd generation) plaque and found slight variations in slot length, width, and depth, but did not indicate any error in the seed center locations obtained from PS.^28^, ^29^ After casting, the thickness of the plaque and the depth of the notches is measured using a micrometer jig with a precision of 0.003 mm and a custom 0.8 mm diameter rounded tip.^27^ Plaques are expected to vary no more than several hundredths of a millimeter between plaque castings. Dosimetric differences resulting from variations in the dimensions of the plaques have been investigated.^28^, ^29^ These studies show that at distances on the order of tumor apices, the difference from small deviations in slot construction is on the order of a few percent, which is not considered to be clinically relevant, and are on the order of similar differences resulting from uncertainties in COMS plaque seed locations.^30^

Manually determining the seed locations of each plaque intended for use for comparison to PS was beyond the scope of this work. Second generation plaques are all cast from the same mold and have been in use for over 30 years with no indication in the literature or results of any issues with seed location beyond a reasonable variation in slot dimension that may result in dose differences that we determined to be not clinically significant. The seed coordinates of third generation plaques in PS come directly from the 3D print files so should be equivalent to the manufactured plaques to within manufacturing tolerances. Therefore, after investigating the literature and comparing the seed locations in PS and in BV, we considered the EP plaques to be available for clinical use pending their successful commissioning in BV.

#### 1.3.2 Plaque commissioning in BrachyVision

**FIGURE 2:**
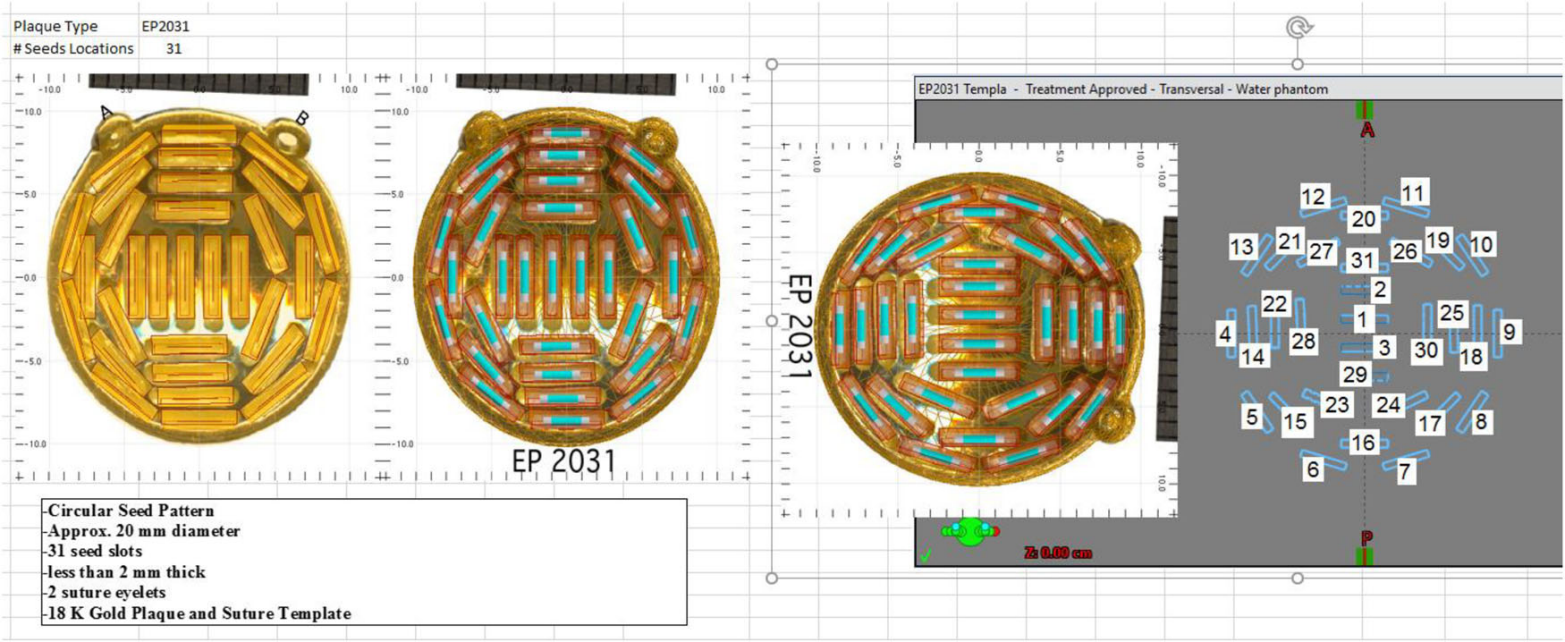
Images of the EP2031 plaque and information from the Plaque Simulator software and numbered seed locations

The first plaques commissioned in BrachyVision (BV) for our program were the EP917, EP517, EP2029N, and EP2031, as these were the first models requested to be used by the ocular surgeon to cover a wide variety of tumor dimensions (see Figure 1). Other plaque models have since been commissioned in BV using this same method, including the circular EP2342 and notched EP2340N plaques for larger tumors, and the circular EP1821 for mid‐sized tumors. The EP917 is a semi‐elliptical plaque with nominal dimensions of 12.4 mm × 14.4 mm and 17 seed positions. The EP517 and EP2031 are circular plaques with nominal diameters of 14.0 and 18.6 mm, and contain 17 and 31 seed positions, respectively. The EP2029N is a notched circular plaque with nominal diameter in the un‐notched dimension of 18.1 mm and 29 seed positions. A commissioning spreadsheet was created for each plaque and excerpts from the commissioning spreadsheet for the EP2031 will be shown as examples of our process. Figure 2 shows some general information about the EP2031 plaque taken from PS as well as the numbering system of the plaque seed locations.

**FIGURE 3:**
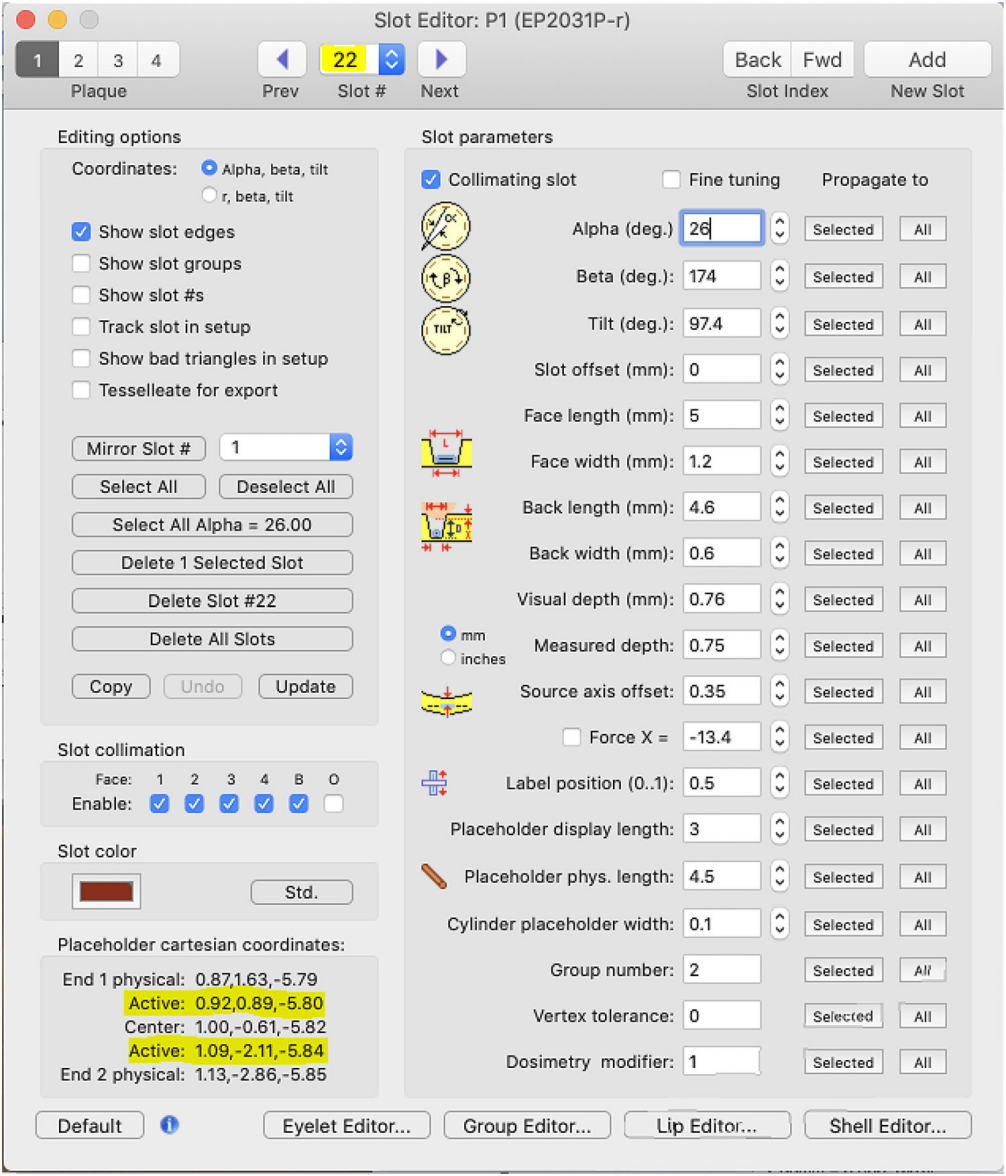
Plaque Simulator Slot Editor window showing the highlighted seed end coordinates for seed slot #22 in an EP2031 plaque. Active lengths were used.

**FIGURE 4:**
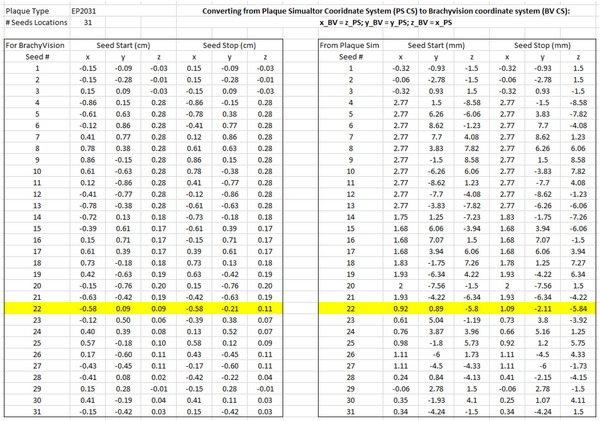
Commissioning spreadsheet excerpt showing conversion of coordinates from Plaque Simulator to BrachyVision. Seed slot #22 is highlighted.

A conversion of coordinates between PS and BV was performed to maintain the z axis in BV as the axis toward the eye center so that it was consistent with the COMS templates previously created in BV. In PS, the x axis is the axis toward the center of the eye. Seed end coordinates of the active length were recorded from the Slot Editor window in the PS software and the PS coordinates were converted into BV coordinates in the following manner: x_BV = z_PS, y_BV = y_PS, z_BV = x_PS. After converting these seed end coordinates from PS, they were entered into BV. Figure 3 shows where the seed end information may be found in the Slot Editor window in PS, and Figure 4 shows the conversion spreadsheet used during commissioning into which the seed end information was entered.

**FIGURE 5:**
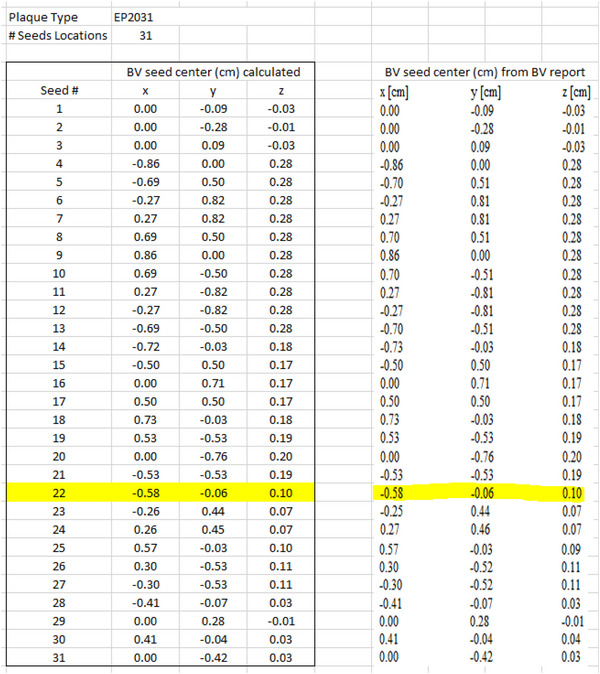
Commissioning spreadsheet excerpt showing the calculation of seed center coordinates and comparison to seed centers shown in the BrachyVision report. Seed slot #22 is highlighted.

For verification of the manual entry of seed end coordinates into BV, seed center coordinates displayed in BV were compared to seed center coordinates calculated in the plaque commissioning spreadsheet. These values in the commissioning spreadsheet shown in Figure 5 varied no more than 0.01 mm due to the rounding of very small physical dimensions of the seeds.

### 1.4 Commissioning Plaque Simulator treatment planning system

#### 1.4.1 Acceptance testing

Plaque Simulator provides a commissioning procedure with expected results to confirm that the software is operating correctly. This involves loading a single 1.0 mCi Amersham 6711 (no longer manufactured) source model into a COMS plaque and confirming certain values and the effect of changing several heterogeneity calculation options. Because we would be using the IsoAid IAI‐125A and not the no longer available 6711 model, after running the tests as specified by Eye Physics, we re‐ran them using the IAI‐125A source model and used those values as the baseline for future annual TPS QA tests.

#### 1.4.2 Configuring plaque simulator software

**FIGURE 6:**
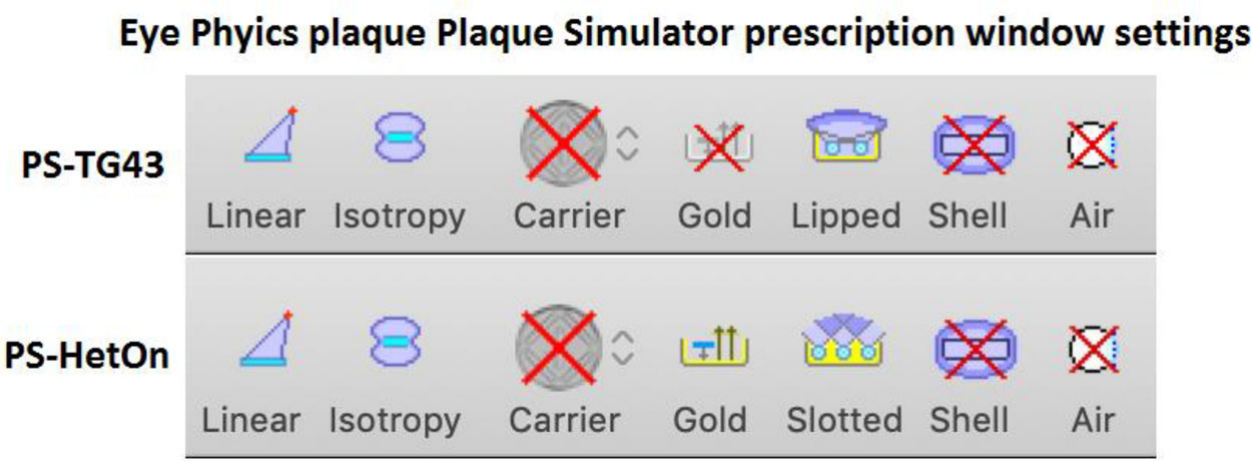
Settings for PS‐TG43 and PS‐HetOn dose calculations for EP plaques

Figure 6 shows the PS settings that can be accessed in the prescription menu that determine how the calculation is performed. There are seven options that may be selected. These options are described below and our settings for either standard TG‐43 calculations or heterogeneity corrected calculations is noted in parentheses. In making selections, follow TG43U^31^ to make sure not to mix point source models with 2D anisotropy corrections.

- Linear/point: selects linear or point source model (enable linear source model for all calculations).
- Isotropy: selects isotropic or anisotropic source model (enable anisotropic source model for all calculations).
- Carrier: applies a correction for COMS Silastic insert (disable for all EP plaque calculations).
- Gold: takes attenuation and fluorescence from gold alloy backing into account (disable for standard TG‐43 calculation; enable for heterogeneity corrected calculation).
- Lipped/Slotted: corrects for plaque specific‐collimation, lipped for COMS plaques, and slotted for EP plaques (select lipped for EP plaques for standard TG‐43 calculation to avoid any correction for the collimating effect of the slot, and select slotted EP plaques for heterogeneity corrected calculations).
- Shell: enables shell collimator ray tracing, which is explicit ray tracing and is a slower calculation and is necessary only for complex plaques with arbitrary shapes since virtually all primary radiation in an EP plaque is collimated at the surface of the slot rather than the edge of the plaque (this is disabled for all calculations).
- Air: takes into account air in front of cornea (because in our flow the standard eye model will be used rather than an eye model based on patient‐specific CT or MR imaging, this function is disabled for all calculations).

#### 1.4.3 Plaque Simulator commissioning of considered Eye Physics plaques

A clinical plan was created in PS for every commissioned EP plaque (EP917, EP517, EP2029N, and EP2031). The activity calculated in PS was then used to calculate the same EP plaque in BV. For each EP plaque model, three plans were generated: (1) a PS plan that turned off heterogeneity corrections (gold off, lipped selected) to result in a standard TG‐43 calculation called “PS‐TG43,” (2) a PS plan that turned on appropriate heterogeneity corrections (gold on, slotted selected) called “PS‐HetOn,” and (3) a BV plan using a standard TG‐43 calculation called “BV‐TG43.” See Figure 6 for the corrections selected.

PS‐TG43 and BV‐TG43 calculations for the same plaques in PS and BV result in dose differences at a point 6 mm away from the surface of the plaque of less than 1.0% for all plaques (EP517 −0.9%, EP917 −0.2%, EP2029N 0.0%, EP2031 0.3%), within the 2.5% recommended as per Table 2 published in TG‐114 on page 511.^32^ This point 6 mm from the inner surface of the plaque, which is equivalent to the location of a tumor apex of 5 mm, was chosen because it is automatically provided by the PS software. This made data collection straightforward and was chosen because that was the point that would be used to check the dose difference when the second check was performed. While 5 mm from inner sclera is a common depth for eye plaque dose comparison calculations, dose differences at different depths could also be investigated.

PS‐TG43 and BV‐TG43 calculations were compared with the PS‐HetOn calculation to quantify differences when accounting for the heterogeneity of the plaque. When comparing the standard TG‐43 calculations in PS or BV to the heterogeneity corrected calculations in PS we see a difference in the range of 5.3%–6.2% (EP517 −5.3%, EP917 −5.9%, EP2029N −6.1%, EP2031 −6.2%), with the standard TG‐43 plans showing a consistently higher dose at the prescription point. The decrease in dose at 6 mm from the plaque seen in our results is on the order of the overall decrease of dose due to backscatter that would be expected, which has been measured experimentally to be on the order of 4% at 5 mm depth for a single seed^33^ and on the order of 8% for a full array of seeds.^34^ The heterogeneity corrections calculated in PS have been studied for the EP917 and the 16 mm COMS plaque. With a single slot loaded of the EP917, the dose determined from Monte Carlo calculations corrected for heterogeneities agreed to within 4.3% with the dose determined from PS calculations corrected for heterogeneities at a depth of 5 mm from the inner sclera.^22^ TG‐221 shows that at a distance of 5 mm from the inner sclera, the difference between Monte Carlo calculations without and with heterogeneity corrections is 7% when calculated with a plaque loaded with 15 I‐125 seeds.^19^ A similar comparison was made using a fully loaded 16 mm COMS plaque and the doses agreed to within 2.4% at a depth of 5 mm from the inner sclera.^35^

After making these comparisons and investigating published work comparing the differences in calculations made both with and without heterogeneity corrections, we considered the PS TPS used with heterogeneity corrections to be commissioned and available for clinical use.

#### 1.4.4 Plaque Simulator eye model, image‐based planning, RDAH/DVH, and prescription considerations

In addition to the various dose calculation methods available in PS, there are also options relating to the dimensions of the eye model used in the calculation. Our process is based on retinal sketches and diagrams provided by the ocular surgeon and does not incorporate fundus imaging or 3D imaging of the patient. For these reasons we chose to use the 24 mm diameter “COMS standard” sized eye model which is the PS default eye model.

PS has extensive functionality for image‐based planning using fundus photographs and ultrasound images as well as 3D imaging (CT and MRI). Our plaque brachytherapy program was not implemented with the addition of image guidance; however, continuing process improvements of the program are being undertaken and image guidance may be added to our treatment planning paradigm in the future. It has been shown that incorporating patient‐specific imaging can be especially beneficial when treating tumors closer than 6 mm to the optic disk.^36^ Implementing registered images and evaluating plan quality based on actual patient anatomy could be of substantial clinical benefit.

Changing from COMS plaques to EP plaques led to a change in the standard prescription dose. As mentioned previously, after retrospectively analyzing the outcomes of studies prescribing different radiation doses with COMS plaques,^15^, ^16^, ^17^ a standard dose prescription of 70 Gy had been in use for a nominal COMS‐style dose that did not account for the attenuation from the Silastic plaque. Because the EP plaques do not use a Silastic insert, we decreased the prescription dose such that the physical dose delivered with the EP plaques would be similar to the dose delivered with the previous COMS plaques. Upon review of the average decrease in dose due to attenuation across the various sizes of COMS eye plaques^18^, ^37^, ^38^ it was determined that the standard prescription dose of 70 Gy should be decreased by 10% to 63 Gy.

**FIGURE 7:**
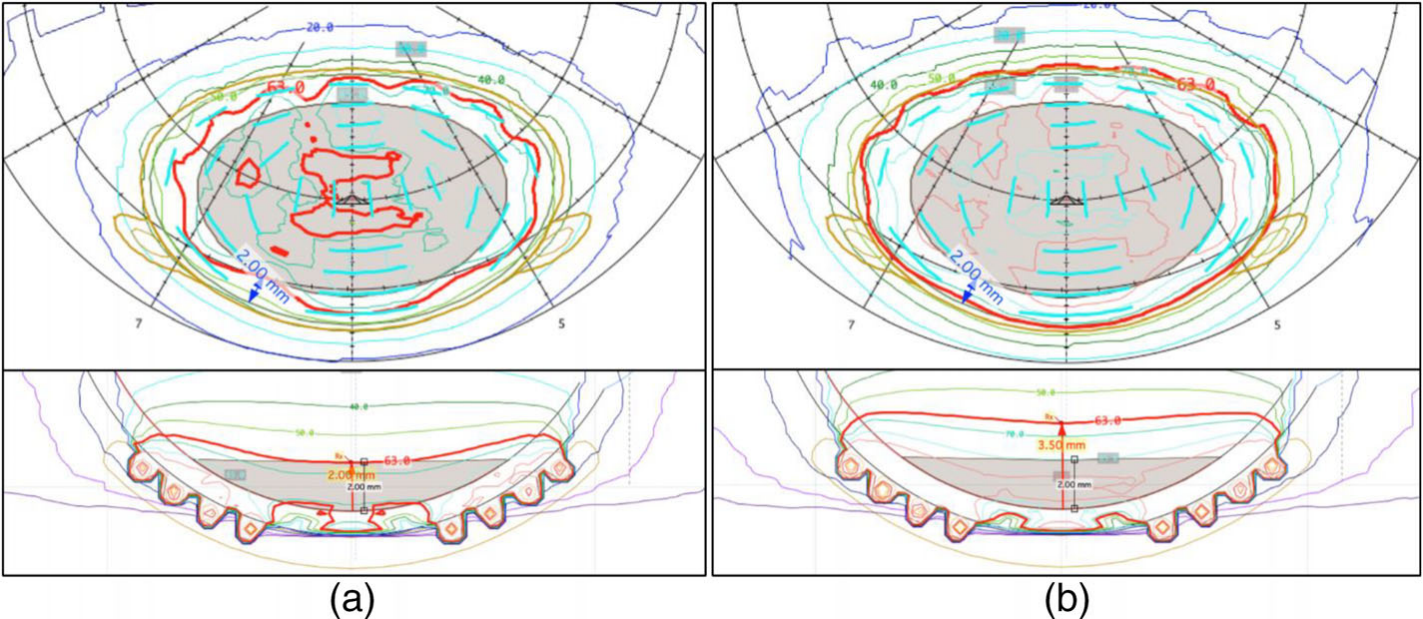
(a) Shows a tumor with an apex height of 2.0 mm prescribed to 2 mm planning apex height. The margin is not covered by the prescription dose. (b) Shows the same tumor prescribed to a 3.5 mm planning apex which results in full margin coverage.

Even without the use of patient imaging, the heterogeneity corrections available in PS allow a more accurate dose calculation that takes into account the attenuation and fluorescence from the gold alloy backing, and corrects for plaque‐specific collimation, which results in more realistic isodose lines. For this reason, we chose to calculate the plan to a prescription apex height sufficient to cover the complete tumor plus a 2 mm expanded margin on the base to a minimum of 63 Gy. Depending on the shape, location, and height of the tumor, and also on the size of the plaque being used, the planning apex may be deeper than the tumor apex in order to cover this margin. This may be especially evident when a tumor with a large base dimension is particularly shallow, or when a tumor encroaches on the optic nerve. In these scenarios, the dose to the tumor apex will exceed 63 Gy. Figure 7 shows how prescribing to the tumor apex might not provide margin coverage. The outcomes from this 63 Gy dose level with this planning paradigm has since been published.^39^

**FIGURE 8:**
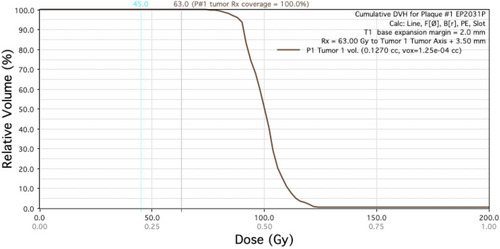
DVH example for the same plan show in Figure 7b

These more realistic dose distributions calculated in PS provides two types of dose histogram structures. The histogram primarily used in plan analysis and comparison is the Retinal Dose Area Histogram (RDAH). This is more useful because most structures of interest in the eye are located in its choroidal and retinal layers. For this reason, a surface area calculation of the inner sclera is more useful than volumetric calculations of the entire eye.^40^ This provides a quick and easy way to optimize seed loading patterns and plaque selection to maintain coverage while also reducing dose to critical structures as much as possible. PS does also provide a traditional Dose Volume Histogram (DVH) that allows verification of coverage of the entire tumor. This histogram is used less frequently during the iterative planning process, but it does allow confirmation that the entire volume of the tumor has been covered by the prescription isodose line. Figure 8 shows the limited utility of such a plot. It indicates that the tumor volume is covered, but since other structures of interest are considered to be 2D and therefore have no volume, they are not included on the DVH.

**FIGURE 9:**
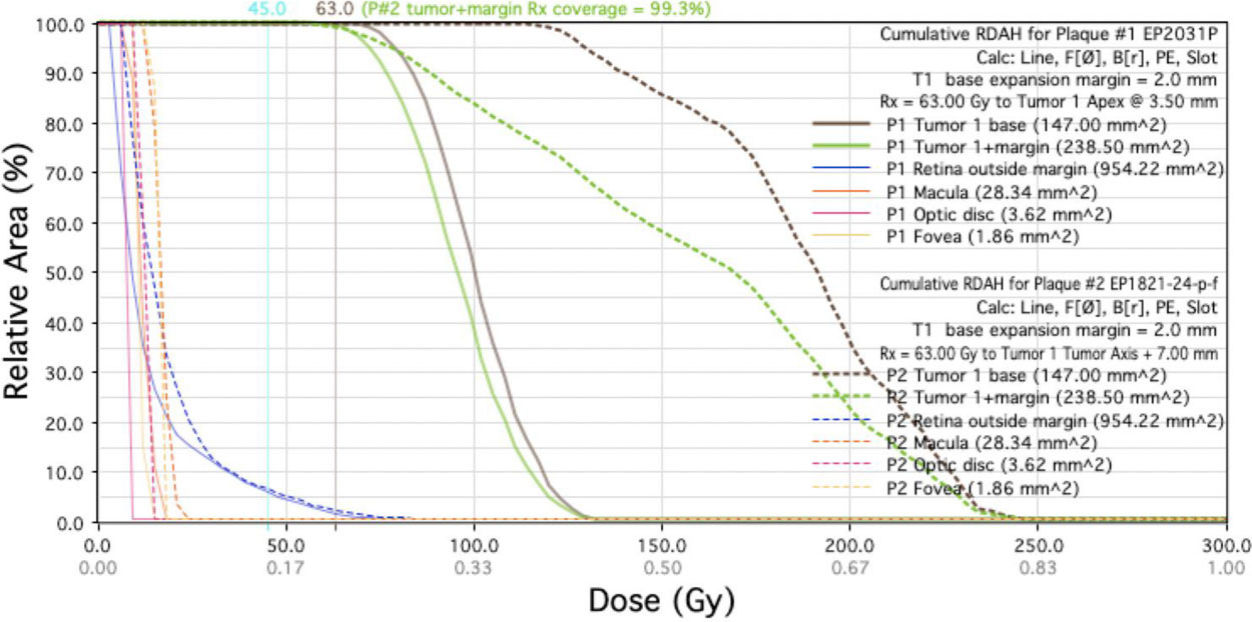
RDAH example comparing two plans for a tumor with base 13 mm × 13 mm and an apex height of 3.5 mm. Plaque #1 is an EP2031 with a planning apex equal to the tumor apex of 3.5 mm. Plaque #2 is an EP1821 plaque (commissioned in 2021) with a planning apex of 7.0 mm.

It is important to note that the RDAH is not used to provide absolute values because in our planning process the tumor shape and distance to normal structures are estimated by the ocular surgeon, and because we use the PS default eye model rather than patient 3D scans. Figure 9 shows how the RDAH may be used to determine the optimal plan. When comparing two plans, the relative coverage of critical structures can be assessed as can the coverage of the tumor and the margin. If image‐based planning were utilized to provide more accurate localization of the tumor relative to the OAR, these RDAH and DVH values could be used directly.

### 1.5 Quality assurance of plaque therapy program

Quality assurance required for developing a plaque brachytherapy program includes establishing an independent second dose calculation check, performing independent dosimetric verification of plaques used for treatment, and creating procedures for continuing program maintenance, upgrades, and improvements.

#### 1.5.1 Establishing an independent second dose check

**FIGURE 10:**
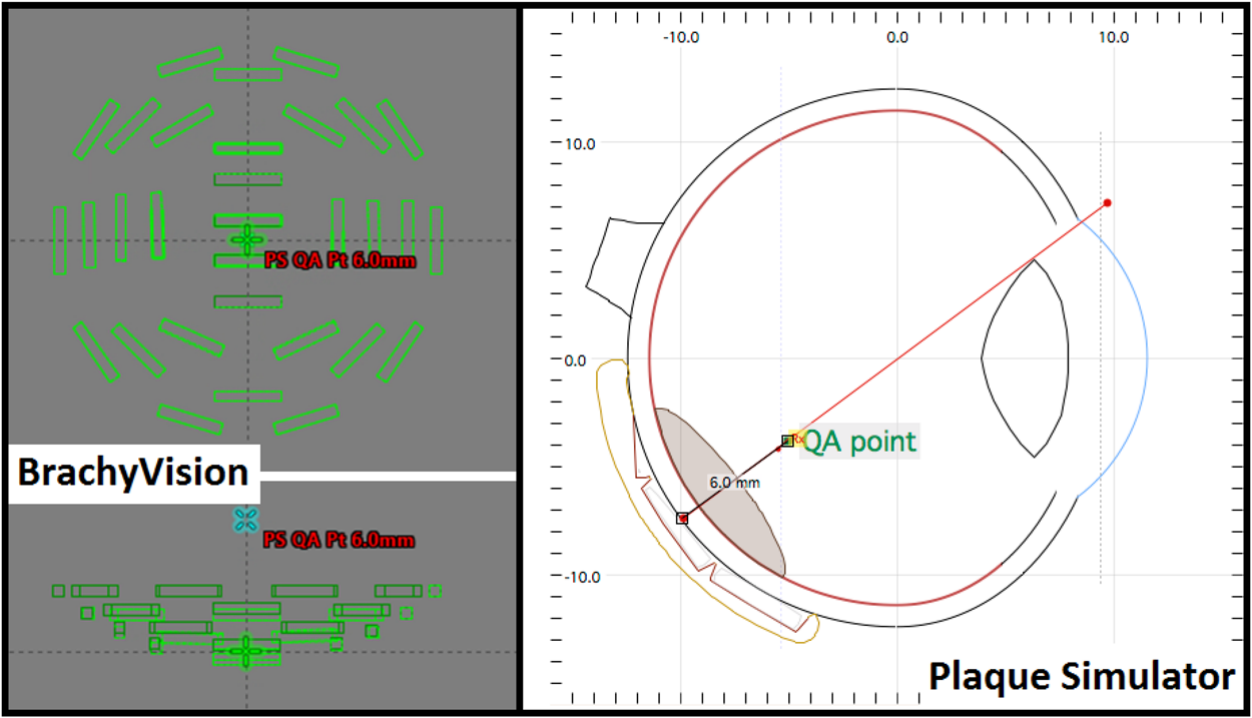
Location of the QA_Point/QA_Check point in BrachyVision and Plaque Simulator

A method was determined to use BrachyVision (BV) Software (VMS) as an independent second check of the PS calculations. PS provides a calculation at a point 6 mm from the inner surface of the plaque called the “QA_Check” point. The QA_Check is a simplified calculation that assumes the seeds are isotropic point sources in water. This calculation is independently coded in PS so that an institution may choose to use this as the only independent second check. However, it is also possible to use this simplified calculation to a point in conjunction with BV to perform an independent second check using an FDA approved TPS. After planning, the seed activity and prescription information from PS is entered into the EP plaque templates created in BV during the commissioning process and the dose at the same location as the QA_Check point is determined. The two independently calculated doses are compared. Figure 10 shows the location of the QA_Check/QA_Point in BV and in PS.

The independent second check comparing the PS QA_Check (point source) value to the BV (line source) value was completed for every commissioned plaque and agreed to within 2.5% (EP517 −2.5%, EP917 −2.0%, EP2029N 0.7%, EP2031 0.7%). Even with this difference between point and line source model approximations, there is still a better than 3% agreement between PS and BV. This independent second check has been performed for each patient planned at our institution and has been found to be better than 2.9% over the course of the program, within the 5% recommended as per Table 3 in TG‐114 published on page 511.^32^ The largest differences in the second check are from the EP517 plaques because that plaque initially showed the biggest difference between the point source (PS QA_Check) and line source (BV) models.

#### 1.5.2 Independent seed assay program and pre‐loaded, pre‐sterilized plaque considerations

EP plaques are commonly ordered pre‐loaded with pre‐sterilized from the manufacturer. A drawback to this technique is that the activity of each seed loaded into the plaque cannot easily be independently verified by the institution performing the treatment as recommended by TG‐221 and the 2008 AAPM working group^19^, ^41^ and as was previously done by our institution when COMS plaques were loaded in‐house.

The manufacturer performs two independent assays, one when the seeds are manufactured and one when they are loaded into the plaques, the results of both are provided with the plaque documentation. It is possible to order a number of non‐sterile loose seeds from the batch manufactured for and loaded into the plaque. TG‐221 recommends 5% of the total order, or five seeds, whichever is fewer, and even allows for the use of a single seed.^19^, ^41^

It was determined that the technique recommended in Table 1 found in the AAMP working group on low energy brachytherapy source calibration when working with sterile applicators would be followed.^41^ Two loose non‐sterile seeds from the lot manufactured are routinely ordered and assayed before treatment. Based on the number of seeds in the EP plaques in use at our institution (17–31 seeds based on initially commissioned plaques), this follows the 5% recommendation. As we are assaying fewer than 10 seeds, it is expected that these assay seeds are within 5% of the average value of the total lot of seeds. If seeds are not within this action level, we would consult with the radiation oncologist as per AAPM recommendations.^41^

There are also several advantages to receiving pre‐loaded and pre‐sterilized plaques and sending them back to the manufacturer after use. Benefits to this technique include a significant decrease in physics time required for assaying all seeds and assembling and disassembling the plaque, which could take up to several hours for a large plaque. Dose to physics staff is decreased due to the decreased number of seeds that must be assayed and by entirely eliminating the plaque loading step. This process also allows the radioactive plaque to remain in the possession of physics until the moment of the insertion, rather than having it unsecured in a hospital sterilizer for any period of time. Finally, sending back the plaques and assay seeds after use eliminates the need to store and maintain an inventory of decaying sources.

#### 1.5.3 Creating procedures for program maintenance, upgrades, improvements, and data accessibility

With respect to continuing updates of the PS software, minor updates are released approximately monthly with major upgrades released less frequently. PS also provides a quality assurance (QA) plan that was designed to check that the software is operating correctly. It is recommended that the test be run after installing an upgrade. This QA plan can also be used as routine annual QA as per AAPM recommendations.^44^ It was determined that only if the updated features are deemed useful to our practice the software is updated. It was also determined that after every upgrade the QA plan provided in the PS TPS would be run and compared to the baseline values from commissioning. Although the QA plan is designed to be used with the model 6711 seed, the IAI‐125A would be the seed used in our program, so the QA plan was run with the IsoAid IAI‐125A seed at commissioning to get baseline values.

End‐to‐end tests of each commissioned plaque are also done after every upgrade. This involves recalculating a standard plan in PS for each plaque. The standard plan consists of a 10 mm diameter tumor with an apex of 5 mm planned to prescription dose at the tumor apex. Seed activity as well as dose to QA point is determined. The same standard plan is run in BV and all values are compared and expected to be within 1% of baseline values.^44^ Our values over the upgrades were the same to within 0.1%.

A decision had to be made how much functionality to allow to the end user in PS. This plaque‐specific program is incredibly powerful, providing the user the ability to modify plaque physical structure and edit physics data. It also allows planning with various isotopes and various seed models of those isotopes. Our institution has a large brachytherapy group, a residency program, and students performing research. It was a concern to us that physics data or plaque geometry might be accidentally changed or modified, or that the wrong plaque or seed model might be used inadvertently. For these reasons, all plaque and physics files that we are not intended to be used clinically were removed from the software's target folder. The only files left was the physics file for the IAI‐125A seed, the EP plaques available for rental and commissioned by our institution, and COMS plaque files for potential future research. A clean copy of all these files was also kept in a protected folder so that any corrupted files could be easily corrected. The use of BV plaque templates, which are difficult to unknowingly edit, as a second check would serve to show if the plaque files were accidentally changed and the clean copies of the plaque files could be used to restore the correct plaque files.

#### 1.5.4 Quality program management

Our quality management (QM) program includes a workflow that includes tasks, documentation, and timeline with the associated responsible team members.^19^ The process map is unique to every institution. A recent FMEA for ocular brachytherapy^45^ for COMS based programs showed that human failure as the highest ranking potential cause of the investigated failure modes and it was the potential cause that was most likely to occur. These include errors by the Medical Physicist in plaque assembly and treatment planning. The transition to a program using pre‐loaded plaques may reduce the likelihood of errors in plaque assembly, especially when coupled with independent dosimetric verification of the plaque.

Our implemented QM methods include onboarding and continuing training, utilizing experienced personnel, and implementing checklists to reduce human failure as a potential cause. In our experience, implementation of training procedures and the use of checklists has been an important feature of the plaque brachytherapy program. Future work may include identifying problems in our program through a retrospective analysis of failures to guide improvements for our quality management program.

### 1.6 Procedures and workflow

Procedures and workflow were established for the use of PS and EP plaques. These were based on our existing procedures and modified as necessary. These guidelines were established for every part of the treatment process starting with the forms used by the eye surgeon to request the plaque and provide tumor dimensions, and proceeding through the entire process through to chart completion. These include procedures for planning, ordering, and creating appropriate patient electronic medical records and written directives. Also included are procedures with radiation safety to ensure proper shipping, delivery and handling. Additional procedures are written outlining the performing of patient‐specific QA, insertion and removal procedures, return shipping of used seeds, and chart completion.

Clinics with active COMS programs may find their established procedures need only minor modifications. For those sites implementing a new plaque program, Appendix A includes examples of documents and checklists created for use in our program.

## 2 SUMMARY

While TG‐221 provides recommendations on commissioning of an ocular brachytherapy program, these recommendations provide only a general summary on steps that a clinical medical physicist needs to perform to commission a program. This may leave out certain detailed considerations we felt needed to be addressed in detail. After years of successful experience with COMS plaques and TG‐43‐based treatment planning, our institution transitioned to an eye plaque brachytherapy program with IsoAid IAI‐125A seeds, Eye Physics plaques, and the Plaque Simulator treatment planning system using BrachyVision as a secondary check. The use of a new seed model, plaque design, and treatment planning system was investigated and a planning and treatment paradigm was successfully developed and commissioned. To better assist other institutions with a similar transition, a step‐by‐step summary of the process we followed to commission our program has been included as Appendix B.

## AUTHOR CONTRIBUTIONS

The authors confirm contribution to the paper as follows: conception and design of the paper: S. Meltsner, A. Rodrigues, O. Craciunescu. Interpretation of results and data, and design of program discussed in the manuscript: S. Meltsner, A. Rodrigues, M. Materin, D. Kirsch, O. Craciunescu. Draft manuscript preparation: S. Meltsner. All authors reviewed the results and approved the final version of the manuscript.

## CONFLICTS OF INTEREST

David G. Kirsch: is a cofounder of and stockholder in XRAD Therapeutics, which is developing radiosensitizers. D.G.K. is a member of the scientific advisory board for and owns stock in Lumicell Inc, a company commercializing intraoperative imaging technology. None of these affiliations represents a conflict of interest with respect to the work described in this manuscript. D.G.K. is a coinventor on a patent for a handheld imaging device and is a coinventor on a patent for radiosensitizers. XRAD Therapeutics, Merck, Bristol Myers Squibb, and Varian Medical Systems provide research support to D.G.K., but this did not support the research described in this manuscript. Miguel A. Materin: Castle Biosciences Advisory Board; Astra Zeneca (Consultant); Carl Zeiss Meditec (Speaker). There is no conflict of interest regarding this manuscript.

## Supporting information

Supporting Information

Supporting Information
