# Supplementary material for: Transitioning from a COMS‐based plaque brachytherapy program to using eye physics plaques and plaque simulator treatment planning system: A single institutional experience
Source: J Appl Clin Med Phys. 2023 Jan 13;24(5):e13902. doi: 10.1002/acm2.13902 (PMC10161060; doi:10.1002/acm2.13902)

**Appendix A**

**Eye Center Worksheet**


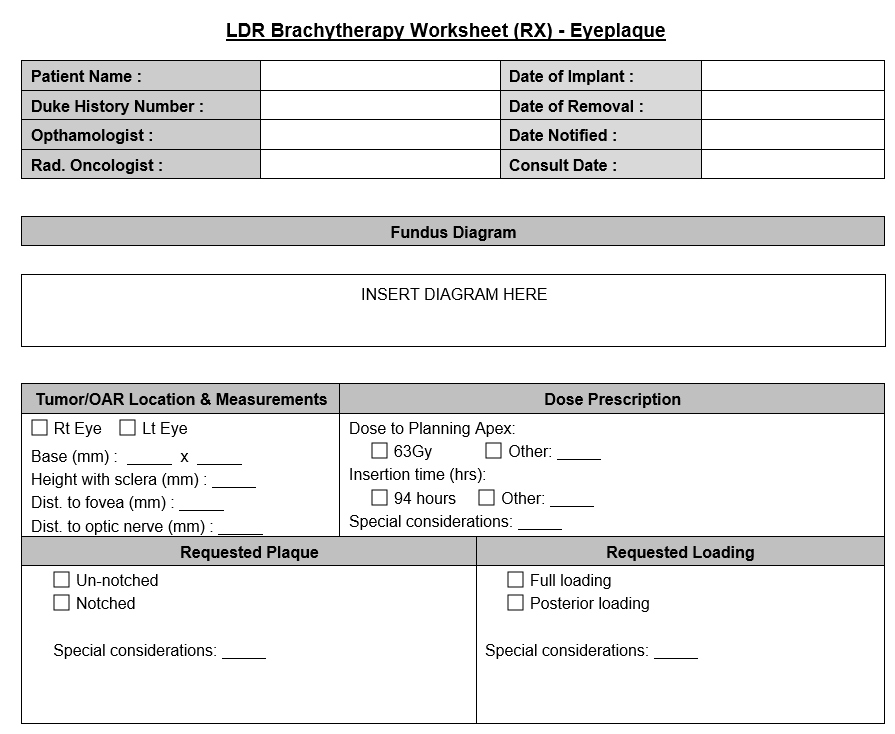


**Planning Folder Checklist**


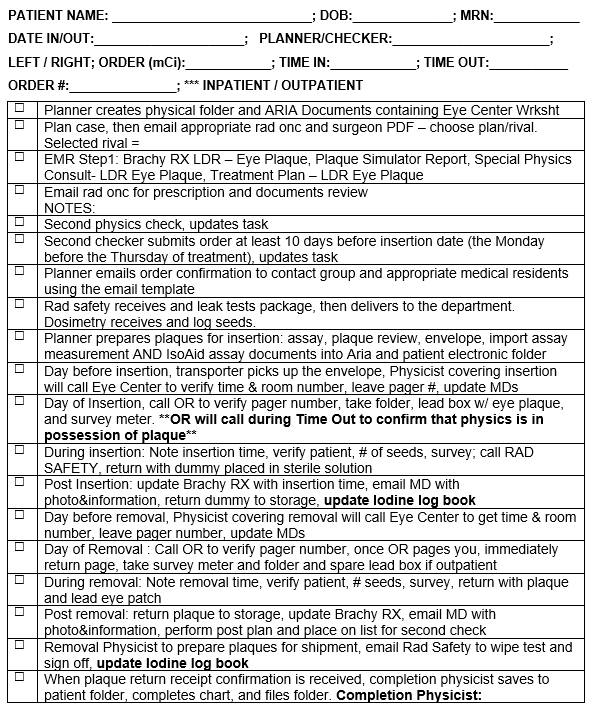


**Patient Envelope Checklist**


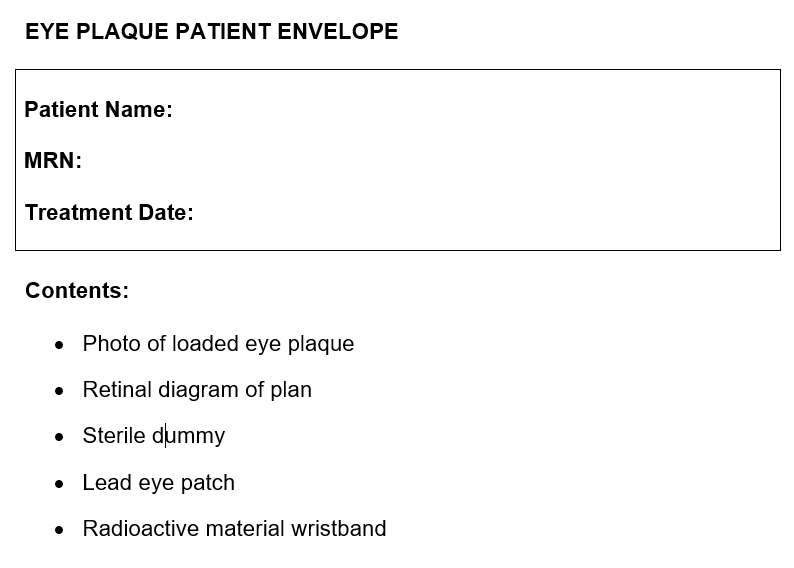


**Seed Assay Document**


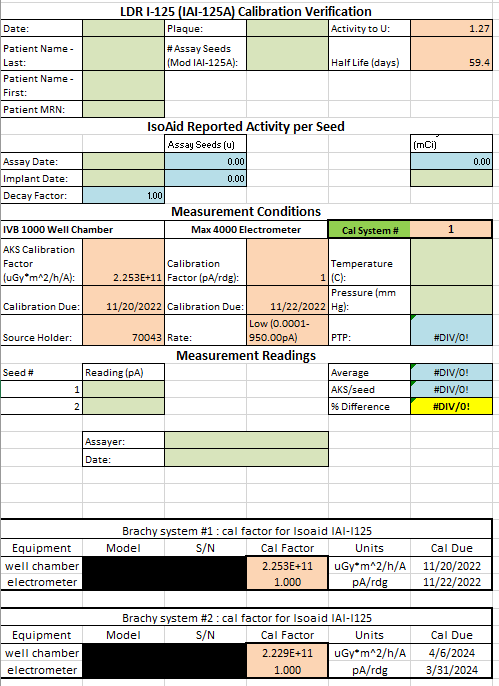


**Insertion Physicist Checklist**


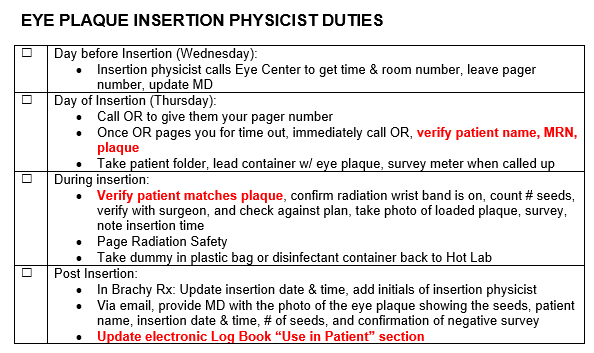


**Removal Physicist Checklist**


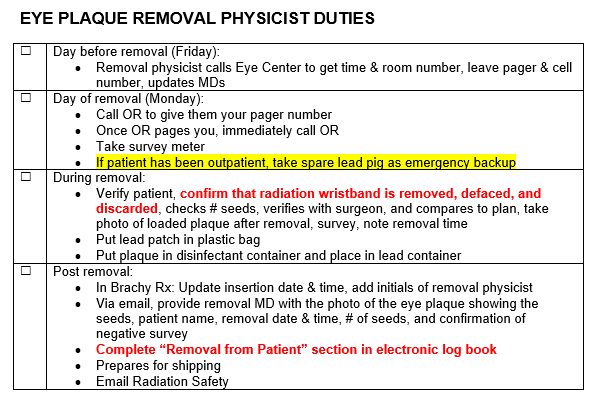

Supplement: Supplementary file 1 — Supporting Information [file ACM2-24-e13902-s001.docx]
