# Supplementary material for: Transitioning from a COMS‐based plaque brachytherapy program to using eye physics plaques and plaque simulator treatment planning system: A single institutional experience
Source: J Appl Clin Med Phys. 2023 Jan 13;24(5):e13902. doi: 10.1002/acm2.13902 (PMC10161060; doi:10.1002/acm2.13902)
Supplement: Supplementary file 2 — Supporting Information [file ACM2-24-e13902-s002.docx]

**Appendix B**

**Step-by-step guide to developing a plaque brachytherapy program using Eye Physics plaques and Plaque Simulator treatment planning system**

1. Address FDA approval of treatment planning system and plaques
   1. Form a panel of senior radiation oncologist(s), medical physicist(s), and ocular surgeon(s) to review your proposal to use Eye Physics (EP) plaques and Plaque Simulator (PS) treatment planning system (TPS).
   2. Present data justifying their use based on the TG-221 stipulation that non-FDA approved devices and TPS may be used if the physicist has taken steps to “appropriately commission and validate dose calculations.” Data may include use in literature and the results of your own commissioning process.
   3. Gain approval for the use of EP plaques and PS TPS in your clinic.
2. Commission IsoAid IAI-125A seed model
   1. If the IAI-125A seed model is not already in use in your clinic, follow your own procedure for commissioning a new LDR seed and commission the IAI-125A seed model in BrachyVision (BV).
   2. Commission the IAI-125A seed model in PS.
   3. Compare isodose lines and point dose values for the same source between both TPS. Do they match to within 2.5%?
3. Commission Eye Physics plaques
   1. Determine which EP plaques will be used in the implementation of your new program and record seed end locations of the slots in those plaques.
   2. Convert those seed end locations found in PS into the coordinate system of BV and enter into BV to create a template of each plaque that will be in clinical use.
   3. Verify these coordinates by comparing seed center locations between PS and BV.
4. Commission primary Plaque Simulator treatment planning system
   1. For each clinically commissioned plaque, generate three plans:
      1. PS plan without heterogeneity corrections (PS Heterogeneity-Off)
      2. PS plan with appropriate heterogeneity corrections (PS Heterogeneity-On)
      3. BV plan using standard TG-43 calculation (BV Heterogeneity-N/A)
   2. Compare PS Heterogeneity-Off plans with BV Heterogeneity-N/A plans. Do they match to within 2.5%?
   3. Compare PS Heterogeneity-On plans to PS Heterogeneity-Off or BV Heterogeneity-N/A plans. Is the magnitude of difference supported by the literature?
5. Create quality assurance plan for plaque therapy program
   1. Establish an independent second dose check
      1. Using all commissioned plaques, create a homogeneity-corrected clinical plan in PS and record the seed activity.
      2. Using the BV templates created in step 3, create a plan using the seed activity determined is step 5.a.1.
      3. Compare the QA_Check dose value from PS to the BV dose value at 6 mm from the inner surface of the plaque. Do they match to within 2.5%?
   2. Independent dosimetric verification of plaques
      1. Order at least two loose seeds from the same lot as is loaded into your clinical plaque.
      2. Using a calibrated well ionization chamber and electrometer, assay these seeds. Do they match the manufacturer’s stated activity to within 5%?
   3. Creating procedures for program maintenance, upgrades, improvements, and data accessibility
      1. Determine how frequently you will update the PS software and what tests you will perform annually and after upgrades.
      2. Determine how much physics data you want accessible to the users of PS and remove any data you don’t want accidentally accessed from PS target folders.
   4. Quality program management
      1. Create your own institution-specific process maps and workflows.
      2. Perform fault tree analysis and failure modes and effects analysis.
6. Create procedures and workflow
   1. Consider the process from start to finish and develop explicit workflows for each part of the process. These may include:
      1. How does the ocular surgeon pass along information about the patient?
      2. At what point does the radiation oncologist consult the patient?
      3. How are plans created, reviewed and approved, and ordered?
      4. What will make up the patient electronic medical record?
      5. How and by whom will the plaques be received and checked in?
      6. How or where will the plaques be stored, assayed, and transported to the procedure room for insertion?
      7. How will the plaques be packaged and shipped back to the manufacturer after the procedure?
   2. Consider the process from start to finish and develop the documents necessary for all departments involved in the procedure. These may include, depending on your specific process:
      1. Ocular surgeon – forms to provide tumor dimensions, sketch or imaging, and specific plaque requests.
      2. Radiation oncologist – written directives, special physics consults.
      3. Medical physics – forms for treatment plan and second check, forms for assay documentation, forms for post planning.
